# The effect of dorsal rim loss on the initial stability of the BioMedtrix cementless acetabular cup

**DOI:** 10.1186/s12917-015-0383-z

**Published:** 2015-03-18

**Authors:** Meredith L Montgomery, Stanley E Kim, Jonathan Dyce, Antonio Pozzi

**Affiliations:** From the Comparative Orthopaedics Biomechanics Laboratory, College of Veterinary Medicine, University of Florida, Gainesville, FL USA; The Ohio State University Veterinary Medical Center, Columbus, OH USA

**Keywords:** Total hip replacement, Dog, Acetabular cup, Dorsal rim

## Abstract

**Background:**

Loss of dorsal acetabular rim (DAR) is a common sequela to canine hip dysplasia. The purpose of this study is to evaluate the effect of DAR loss on the initial stability of the cementless (BFX) acetabular cup. BFX cups were implanted into foam blocks reamed to resemble acetabulae with simulated 0, 25, 50, and 75% DAR loss. Models were tested in edge loading of the lateral surface of the cup with an indenter, and in centered loading with an articulated femoral prosthesis. Additionally, cups were implanted into paired cadaveric canine hemipelves with either no DAR depletion, or removal of 50% of the DAR, and acutely loaded to failure with an articulated femoral prosthesis.

**Results:**

Mean load measured at 1 mm cup displacement during edge loading was not significantly different in foam blocks with loss of 0, 25, 50, and 75% DAR (360 ± 124 N, 352 ± 42 N, 330 ± 81 N, 288 ± 43 N, respectively; P = 0.425). Mean load to failure with centered loads was greatest in blocks with 0% DAR loss (2828 ± 208 N; P < 0.001), but was not significantly different between 25, 50, and 75% DAR loss (2270 ± 301 N, 1924 ± 157 N, 1745 ± 118 N). In cadaveric testing, neither mean load to failure (P = 0.067), stiffness (P = 0.707), nor energy (P = 0.228) were significantly different in control hemipelves and those with 50% depletion of the DAR. Failure in all acetabulae occurred due to acetabular bone fracture at forces in supraphysiologic ranges.

**Conclusions:**

BFX cup stability under normal physiologic loads does not appear to be compromised in acetabulae with up to 50% DAR loss.

## Background

Total hip replacement (THR) is a highly effective treatment option for a variety of hip disorders in dogs. Cementless fixation of THR differs from traditional cemented THR, as the surface texture of the implants allow durable osseointegration of the bone-implant interface [1]. The initial press-fit should provide the early implant stability required for osseointegration. With the BioMedtrix Biologic Fixation (BFX) Total Hip System, initial stability is achieved by impacting the implants into a prepared bed that is marginally smaller than the implants themselves.

Initial stability of the acetabular cup is likely dependent in part upon the quality and quantity of the local bone stock. The dorsal acetabular rim (DAR) may play a particularly important role in the stability of the acetabular cup, as weight bearing forces are concentrated on the DAR in a canine hip [2-4]. Attrition of the DAR due to chronic subluxation of the femoral head is a common finding in dogs with severely dysplastic hips [2]; it is possible that this pattern of bone loss could compromise cup stability. Cemented THR have relied on the DAR for adequate fixation and force dispersal along the acetabulum [5]. As DAR erosion is thought to be an important cause of instability of implants in cemented THR, augmentation of the DAR using several techniques have been described [6,7].

Aseptic cup loosening has been reported in cases undergoing cementless THR, although there is little information specifically pertaining to complications with the BFX cup [5,8]. This cup was designed to predominately rely on a cranial to caudal press fit, whereby the early stabilizing forces are thought to be dependent on the integrity of the cranial and caudal acetabular margins^a^. The current guidelines for implanting this cup indicate that complete coverage by dorsal acetabular bone stock is not a prerequisite, as substantial DAR loss may not be the primary determinant of implant-bone interface failure^a^.

To our knowledge, the mechanical stability of the BFX cup has not been evaluated in the face of poor DAR integrity. The purpose of this investigation was to assess the biomechanics of the BFX cup-bone construct with loss of DAR coverage. The null hypothesis was that initial cup stability would not be significantly affected by DAR loss.

## Methods

This study consisted of two phases. The first phase of the study was conducted in polyurethane foam blocks with incrementally increasing loss of simulated DAR. This model was developed based on previous in vitro testing of human acetabular implants in polyurethane foam blocks [9,10]. The second phase of the study was performed in canine cadaveric specimens. This study was approved by the University of Florida Institutional Animal Care and Use Committee (#201207313).

### Foam block testing

**Figure 1:**
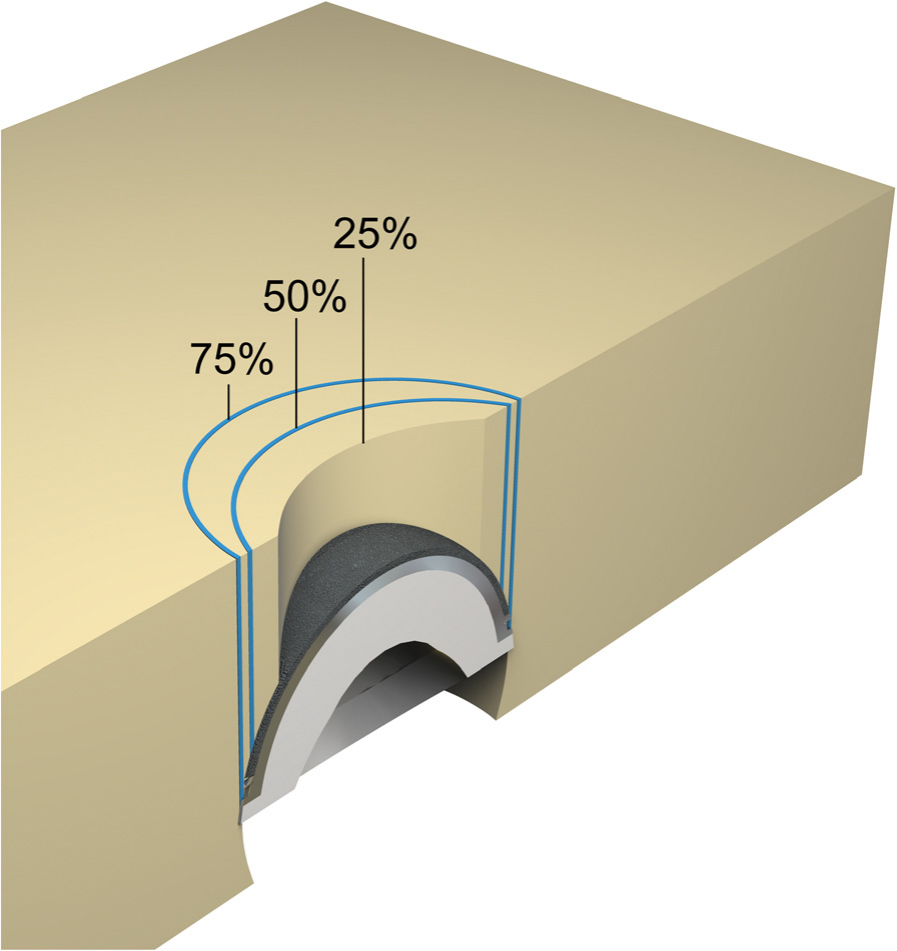
**Illustration of a BFX acetabular cup implanted into a polyurethane foam block with 25% loss of simulated dorsal acetabular rim.** Curved lines represent the margins of 50% and 75% dorsal acetabular rim loss.

The polyurethane foam blocks were prepared to simulate a canine acetabulum. A 23 mm diameter acetabular preparation bed was reamed into solid, homogenous polyurethane blocks (Sawbones Inc., density: 0.4 g/cm^3^) (16 × 7 × 5 cm) using BFX reamers mounted in a standard drill press operating at 500 r.p.m. The foam blocks were sequentially reamed with size 18, 21, and 23 BFX cutting reamers, followed by a size 24 BFX finishing reamer to a depth of 16 mm to simulate an acetabular preparation bed. Reaming, which was performed coaxial with the planned axis of cup insertion, was oriented so that a simulated 10 mm dorsal acetabular rim was preserved. Blocks were assigned to 0, 25, 50, and 75% DAR loss groups (n = 6 per group). For simulating DAR loss, the blocks were oriented in a ‘dorsal’ position under BFX reamers mounted to a drill press for creation of a cylindrical recess. All DAR loss was modeled as a curved recess and was calculated as a percentage of the depth of the simulated acetabulum (25% = 4 mm, 50% = 8 mm, 75% = 12 mm DAR loss) (Figure 1). Block stabilization and cylindrical reaming of the DAR was used to preserve simulated cranial and caudal acetabular margins for press-fit stability. Cups were manually impacted into polyurethane blocks by a board certified surgeon (S.E.K) with a force replicating cup impaction in the clinical setting. The cups were oriented and stabilized at a 45° ‘lateral opening’ angle, and 0° ‘version’ and ‘inclination’ angle. For all specimens, the cups were confirmed to be appropriately seated, with the lateral truncated margin of the cup recessed by 1 mm relative to the surface of the block at the cranial and caudal poles of the cup [11]. Four cups were available for all foam block testing. Between each experiment, the cups were manually cleaned until all grossly visible debris was removed.

**Figure 2:**
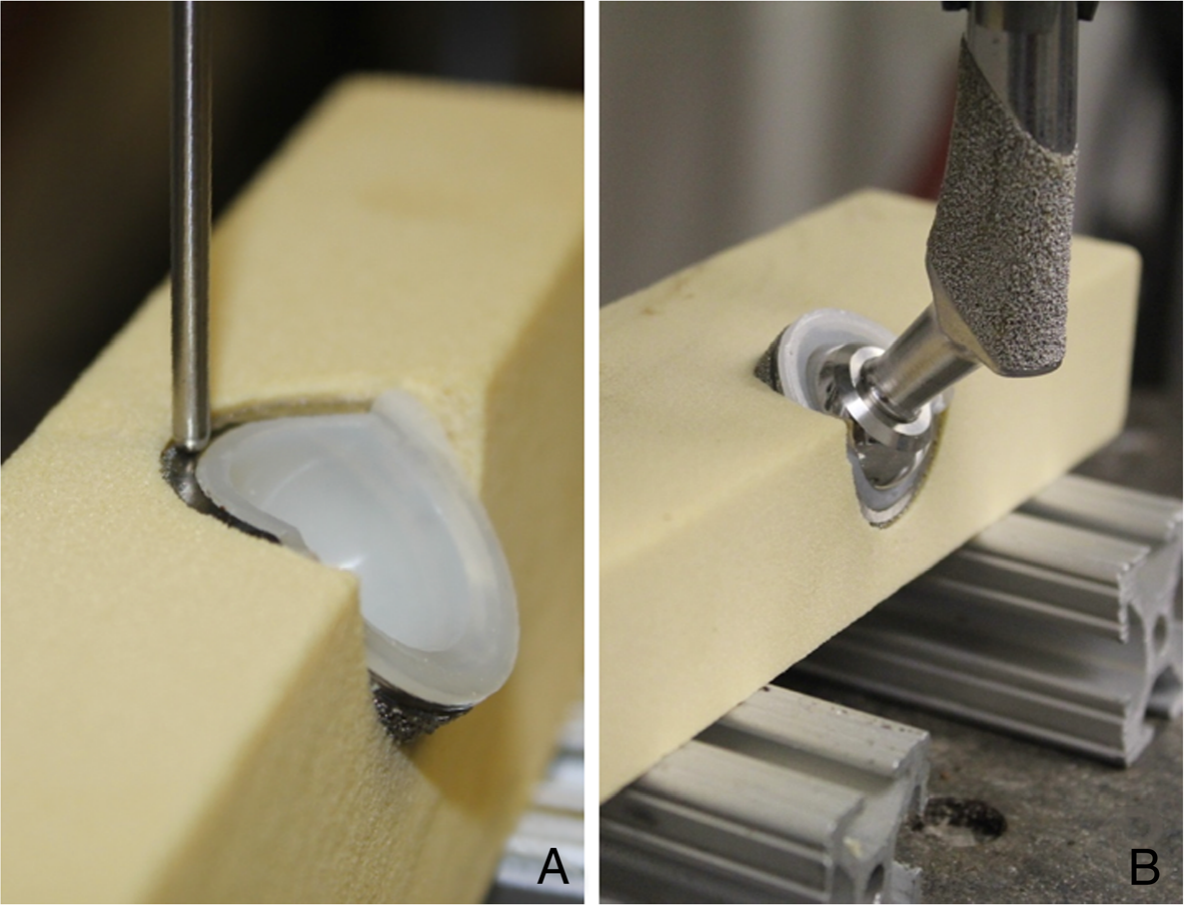
**Acetabular prosthesis – polyurethane block constructs axially loaded with a metallic indenter for edge loading (A), and prosthetic femoral head and stem (B).**

Mechanical testing was performed with a materials testing machine (MTS Corporation) with the foam blocks resting on a custom-made platform. Two patterns of loading were performed: 1) edge loads applied with a metallic indenter (Figure 2A), and 2) centered loads applied with a BFX femoral stem and head (Figure 2B). For edge-load testing, the cup-block unit was oriented with the ‘lateral’ side facing up, and a ramping load under displacement control was applied with the indenter to the center and most dorsal aspect of the truncated lateral face of the acetabular cup at 1 mm/sec. Data was acquired at failure, which was defined in our setup as 1 mm of cup displacement as measured by the materials testing machine actuator. For centered testing, the cup-block unit was oriented with the ‘ventral’ side facing up. A potted #7 BFX femoral stem with 17 mm BFX head was secured to the actuator of the materials testing machine. The femoral head was seated into the polyethylene lining of the cup, and used to apply a ramped load under displacement control at a rate of 1 mm/sec until failure. Failure was defined as gross cup dislodgment or block fracture. All data was acquired from the materials testing machine software.

### In vitro cadaveric testing

**Figure 3:**
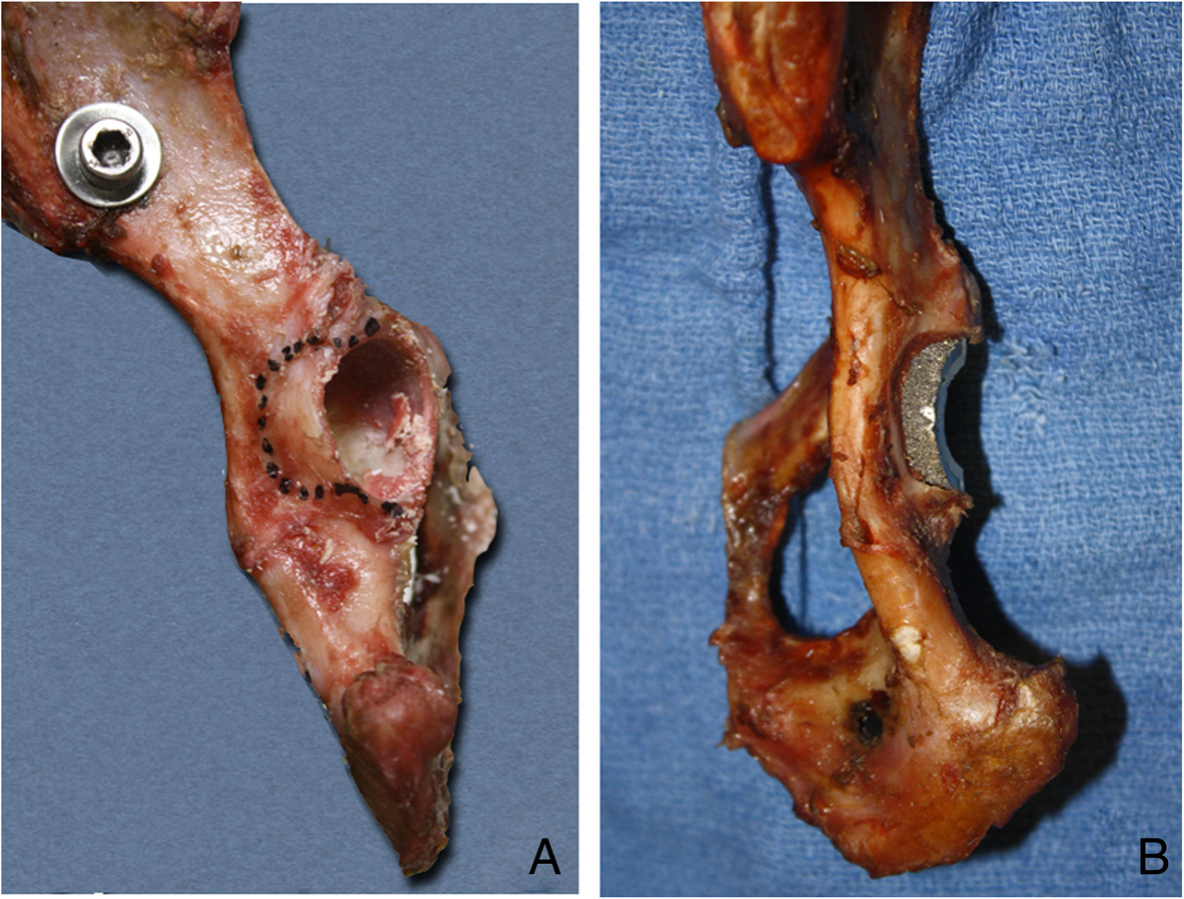
**A hemipelvis specimen with the level of dorsal acetabular rim resection outlined with a permanent marker (A), and after dorsal acetabular rim resection and impaction of the BFX acetabular prosthesis (B).**

Eight mature, mix-breed canine cadaveric pelves (from dogs of 22–28 kg body weight) were radiographed and templated according to manufacturer’s guidelines^a^. All acetabula had dimensions suitable for a 24 mm BFX cup. After removal of the soft tissues, the pelves were divided into hemipelves along the pubic symphysis and the sacrum. The specimens were wrapped in saline soaked towels, sealed in plastic bags and frozen at −20 C until testing. The specimens were thawed to room temperature 24 hours prior to testing. Each pelvis had left and right hemipelves randomly assigned to a normal (control) or DAR loss group. The hemipelves were rigidly secured with 6 mm bolts and nuts to a custom jig that was used for cup implantation and mechanical testing. With each specimen oriented lateral side facing up, a 23 mm diameter acetabular preparation bed was reamed and a 24 mm BFX cup was implanted by a board-certified surgeon (S.E.K) according to manufacturers guidelines^a^. An alignment rod was used during reaming and cup insertion to allow for consistent cup orientation. A bony landmark method^a^, with relation to the ilium, ischium, and pubis, was also used to aid proper cup orientation. Cups were placed with a 45° lateral opening angle, 10° of retroversion, and 10° of inclination^a^. For hemipelves assigned to the DAR loss group, 50% of the dorsal rim was removed with a high-speed burr prior to reaming and cup impaction. This loss was approximated as a curved recess preserving the cranial and caudal acetabular margins, and extending up to (but not involving) the ischiatic spine (Figure 3A). This degree of DAR loss resulted in approximately 7 mm of cup exposure dorsally after impaction (Figure 3B). Three BFX cups were used in cadaveric testing and thus had to be re-used; debris was removed with water and a soft toothbrush, and dried between testing.

**Figure 4:**
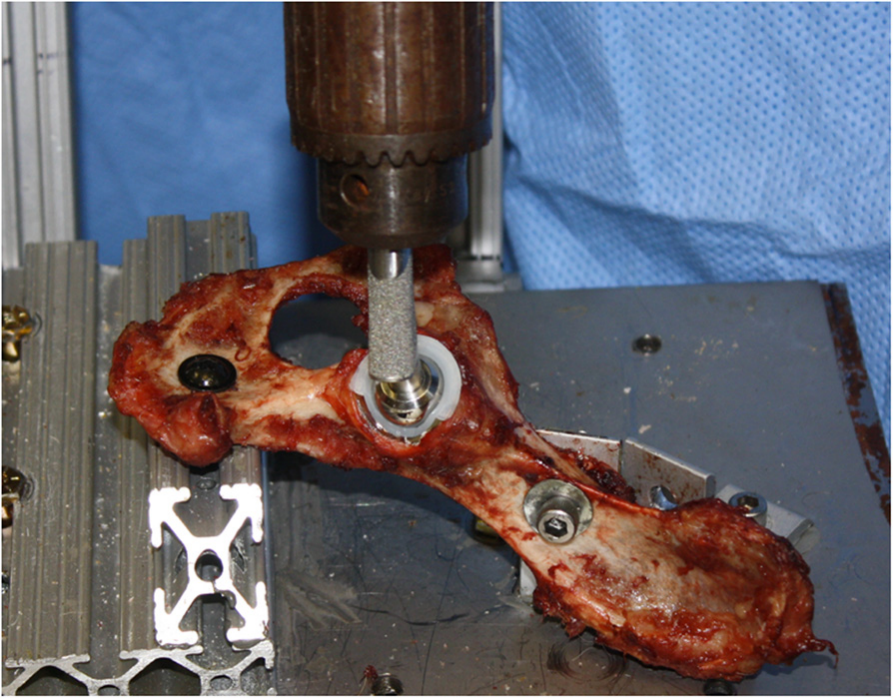
**Specimen testing set up of the hemipelves with implanted acetabular cup loaded to failure at a standing angle using a prosthetic femoral head and stem.**

Each hemipelvis was mounted in a testing fixture and a #7 BFX femoral stem with a 17 mm BFX head was secured to the actuator of the materials testing machine to load the acetabular cup (Figure 4). The pelvis was oriented to replicate a standing angle relative to the femoral stem and head during testing, with 15° of femoral neck anteversion, 0° of abduction, and 110°of hip extension [3]. Pelvic orientation relative to the femoral stem was visually set with a goniometer. Axial loads were applied to the cup with the femoral prosthesis under displacement control at 1 mm/sec until failure. All cadaveric trials were video recorded to evaluate the cause of bone-cup interface failure, which was classified as either gross cup dislodgment or bone fracture.

### Statistical analysis

For the polyurethane foam block experiments, load at failure for centered load testing, and load at 1 mm of displacement for edge load testing were compared between control and DAR loss groups using one-way ANOVA with a post-hoc Tukey HSD for pairwise comparisons. For the cadaveric testing, load at failure, stiffness, and energy were compared between control and DAR loss groups using paired t-tests. *P* < 0.05 was considered statistically significant.

## Results

Mean ± SD load at failure of polyurethane foam block testing are summarized in Table 1. In edge load testing, DAR loss of 25, 50, and 75% resulted in a mean 2%, 8% and 22% lower load at 1 mm of displacement when compared to 0% DAR loss, respectively; however, these changes were not significantly different (P = 0.425). Mean load at failure with centered loads was greatest in blocks with 0% DAR loss (P < 0.001). Loss of 25, 50, and 75% of DAR resulted in a mean 20%, 32% and 38% decrease in load to failure, respectively. There was no significant difference in load at failure for centered testing between groups with 25, 50, and 75% of DAR loss. Failure for centered testing was always due to block fracture at the level of the cup. Results of cadaveric testing are summarized in Table 2. Neither mean load to failure (P = 0.067), stiffness (P = 0.707), nor energy (P = 0.228) were significantly different in control hemipelves and those with 50% depletion of the DAR. Failure in all cadaveric specimens was manifest as acetabular bone fracture at supraphysiologic forces.

**Table 1:** **Mean ± SD edge load to 1 mm displacement of acetabular cups and centered load to failure in foam block testing**

**Group**	**Mean ± SD edge load at 1 mm displacement (N)**	**Mean ± SD centered load at failure (N)**
0% DAR loss	359 ± 124	2829 ± 208^a^
25% DAR loss	351 ± 42	2270 ± 301^b^
50% DAR loss	330 ± 81	1924 ± 157^b^
75% DAR loss	281 ± 42	1745 ± 118^b^
P value	0.425	0.001

Values with different superscript letters are significantly different from each other.

**Table 2:** **Mean ± SD centered load to failure in cadaveric hemipelves**

**Group**	**Stiffness (N/mm)**	**Load at failure (N)**	**Energy (N × mm)**
0% DAR loss	518 ± 206	3592 ± 661	14458 ± 5605
50% DAR loss	570 ± 200	2946 ± 316	10875 ± 3921
P value	0.707	0.067	0.228

## Discussion

Our study utilized two ex-vivo methods of evaluating the integrity of the BFX cup – bone interface. Both cadaveric specimens and polyurethane foam blocks with similar mechanical properties to cancellous bone [12] were used. The resistance generated between the cup and foam block with edge loading was not affected by removal of up to 75% of the simulated DAR region. When tested under axial compression with the femoral prosthesis, all levels of DAR loss failed under loads that were in the supraphysiologic range (2510–4640 N) in both the cadaver specimens and foam blocks. These values were 12-23x larger than expected force during normal daily activity [4,13]. According to our ex-vivo data, it would appear that DAR loss levels typically observed in dogs with hip dysplasia do not significantly compromise BFX cup stability.

One major question to consider when testing press-fit cups is what failure mode is being represented. Our study did not attempt to test the effect of DAR loss on cup micromotion. Greater micromotion of the BFX cup may be present in acetabula with poor DAR, which could result in a fibrous interface and predispose to aseptic loosening, a complication seen in cemented canine THR [14]. Rather, both rim loading and centered loading tests simulated acute loosening accompanied by gross movement of the implant; thus our findings only give insight into initial cup stability. Further studies on canine acetabular cup micromotion are required to elucidate mechanical causes of aseptic loosening. Cyclic testing using a similar testing set-up may provide further insight into cup stability beyond the very early post-operative period.

While it would have been preferable to test varying degrees of DAR loss for the cadaveric component of the investigation, we elected to assess only one magnitude, 50% loss, for several reasons. The lack of significant difference between the 25, 50, and 75% loss in both edge and centered foam block loading suggested that the biomechanical consequences over a wide range DAR loss were roughly equivalent. Also, 50% DAR loss with our method of resection subjectively appeared to most closely replicate poor acetabular conformation in dogs with severe hip dysplasia. It is possible that removal of more than 50% DAR in the cadaveric specimens would have caused failure within a physiologic range of limb loading; however, a defect extending into the isciatic spine seemed to resemble far less common bone morphology, such as acetabular fracture malunion.

Robust stability despite partial loss of DAR can be explained by the design of BFX system, where the press-fit mechanism is thought to be predominately generated between the cranial and caudal acetabular margins. The most important factor in determining press fit cup stability in human THR is the ability of the cup to engage bone around the entire outer periphery [15]; however circumferential rim contact is not possible in dogs because the normal canine acetabulum is not hemispherical. Although we did not specifically test the contributions of the cranial or caudal acetabular margins to cup stability, our results suggest that the predominating press-fit mechanism of the BFX cup is achieved cranio-caudally rather than dorso-ventrally or circumferentially, as typically described for human THR systems.

Medialization of the cup by deeper reaming through the medial wall has been advocated for acetabula with poor DAR coverage, with the goal of increasing dorsal coverage of the acetabular cup [16]. Our results suggest that this approach may not be necessary, as we suspect the loads at failure were not compromised by loss of DAR to a clinically significant degree. In a study of medially displaced BFX cups in normal canine hemipelves [17], loads at failure were comparable to the loads observed in our cadaveric testing, both of which were well above the physiologic range. Based on these findings, we do not routinely advocate reaming through the medial wall as long as the cup is well engaged between the cranial and caudal acetabular margins, regardless of DAR bone stock.

There were several additional limitations to our study. Our DAR resection did not replicate microstructural changes that occur during remodeling with hip dysplasia; however, we suspect subchondral sclerosis observed with degenerative joint disease would have provided even more support than the normal porous cancellous bone. We were required to re-use cups throughout all testing. Debris was removed and no gross deformation of the cup or polyurethane lining was apparent, nor were any decreasing trends in total load to failure apparent in successive testing and reuse of the cups. Nevertheless, repeated cup use may have subtly damaged the porous surface and it would have been ideal to use new cups for each individual test. Cobalt-chromium bead-sintered BFX cups were used, and the results may not be directly applicable to the newer electron-beam-melted titanium BFX cups that are now commercially available. We attempted to standardize the force applied to the impactor during cup placement; however it was subjectively deemed that manual cup impaction as performed in a clinical setting was a more consistent method during pilot testing. Because we were unable to ensure identical impact strength for each specimen, it is possible that some inter-specimen variability was due to slightly inconsistent cup impaction. Mild variation in cup positioning may have also contributed to some variability. All cadaveric specimens failed by fracture of the acetabulum rather than cup dislodgement. It is conceivable that our testing set up was designed so that bone fracture was the only mechanism of failure, which very rarely occurs in clinical cases [18]; however, we utilized a set up for assessing canine THR cup biomechanics [17] where failure caused by cup dislodgement was observed. Finally, there are inherent limitations using displacement values that were required for estimating specimen stiffness from the materials testing machine, due to deformation of acetabular cup, femoral prosthesis, bone, and jig that were not individually quantified.

It is also prudent to note that while adequate cup stability may be attained in dogs with loss of DAR, other factors may contribute to cup loosening and dislodgement. In the clinical setting, intraoperative cup stability may be compromised by a number of technical errors, such as soft tissue entrapment, wobble during reaming causing inappropriate expansion of the acetabular bed, poor reaming alignment, and failure to deliver the cup coaxial to the reamed bed. To the authors’ knowledge, there is only one report of cup dislodgment with the BFX system [18]. In this case, inadequate cup stability was attributed to poor DAR bone stock and subsequent DAR fracture; however, post-operative radiographs suggested that reaming and cup placement was centered dorsally over the DAR itself. Thus in addition to lack of DAR, poor reaming alignment would have placed the cranial and caudal poles of the cup relatively dorsal to the cranial and caudal acetabular margins, which may not have generated appropriate craniocaudal press-fit. Our results may only apply to when there is precise execution of acetabular bed preparation and cup impaction.

## Conclusion

Overall, our study corroborates clinical observations that the BFX cup can be safely used in dogs with DAR loss due to hip dysplasia. DAR augmentation or medialization of the cup may not be necessary, so long as cup implantation performed correctly and sufficient press-fit between the cranial and caudal acetabular margins is achieved. Larger scale investigations correlating DAR loss with the risk of cup related complications are required for more definitive evidence that DAR loss is not a clinically relevant factor for dogs undergoing BFX THR.

## Endnotes

^a^BioMedtrix Inc. Canine Modular Total Hip Replacement System, Surgical Protocol for BFX™ Cementless Application, BioMedtrix Inc., Boonton, NJ.
